# The Metabolism of *Leuconostoc* Genus Decoded by Comparative Genomics

**DOI:** 10.3390/microorganisms12071487

**Published:** 2024-07-20

**Authors:** Francesco Candeliere, Laura Sola, Enrico Busi, Maddalena Rossi, Alberto Amaretti, Stefano Raimondi

**Affiliations:** 1 Department of Life Sciences, University of Modena and Reggio Emilia, 41125 Modena, Italy; francesco.candeliere@unimore.it (F.C.); laura.sola@unimore.it (L.S.); enrico.busi@unimore.it (E.B.); maddalena.rossi@unimore.it (M.R.); 2 Biogest-Siteia, University of Modena and Reggio Emilia, 42124 Reggio Emilia, Italy

**Keywords:** *Leuconostoc*, comparative genomics, functional genomics, metabolic reconstruction

## Abstract

*Leuconostoc* encompasses a number of species that frequently appear in foods where they play different roles, ranging from ripening to spoiling. The number of available *Leuconostoc* genomes has recently increased and enabled the precise taxonomic and phylogenetic delineation of species. Nonetheless, a thorough investigation of the functions and the metabolic potential of *Leuconostoc* species has never been accomplished. In this study, all the currently available 553 *Leuconostoc* genomes were downloaded from NCBI GenBank and annotated utilizing specific tools in order to reconstruct the metabolic potential of the genus in terms of carbohydrate hydrolysis and fermentative pathways, transporters, and anabolic potential. The analysis revealed that species cluster based on their metabolic potential, showing unique adaptation and ecological roles. Pentose phosphate and phosphoketolase pathways were highlighted as the main ones of central metabolism. The various identified PTS and ABC transporters showed adaptability to different sugars. The metabolic diversity described in this study not only supports the role of *Leuconostoc* spp. in natural ecosystems but also highlights their potential in industrial applications, particularly in the fermentation industry where their ability to metabolize a wide range of substrates can be harnessed for the production of various fermented foods and bioproducts.

## 1. Introduction

The genus *Leuconostoc* includes Gram-positive, catalase-negative, facultative anaerobic bacteria, distinguished by their unique metabolic capabilities among heterofermentative lactic acid bacteria (LAB) [1,2]. These bacteria have complex nutritional needs, requiring amino acids, peptides, nucleic acid derivatives, vitamins, fatty acids or their esters, and fermentable carbohydrates [3]. Typically, *Leuconostoc* species inhabit plants, but they are also found in nutrient-rich environments like milk, meat, food matrixes, and fermented products [4,5,6]. Some species are notable for their ability to survive at refrigerator temperatures and to thrive at 10 °C, to tolerate 50 g/L salt, and to grow at pH values above 4.5 [7,8]. During fermentation of diverse carbohydrates and substrates, they produce CO_2_, flavor compounds and exopolysaccharides (EPS) [9,10].

*Leuconostoc* species exhibit a broad range of metabolic functions and distinctive phenotypic traits, making them relevant in many human activities. They frequently appear in foods where they play different roles [11,12,13,14]. Leuconostocs may act as starters in fermentation processes to enhance organoleptic properties or serve as bioprotective agents against other spoilage bacteria. On the other hand, they may also contribute to food deterioration, participating in spoilage and impairing product quality. Their widespread presence in foods has significant implications for human health and safety. Most of the representatives from genus *Leuconostoc* have been designated as “generally recognized as safe” (GRAS) [15], affirming their safety for use in food production. However, some clinical cases of human infections caused by this microorganism have been documented, suggesting opportunistic pathogenic behavior. Most of these cases involved severely immunocompromised patients, with no direct link established between *Leuconostoc* isolation and fermented food consumption [16,17,18]. Beyond the food industry, *Leuconostoc* spp. have been utilized in the formulation of innovative cosmetics through bioactive extracts obtained from fermented vegetables [13]. Recently, *Leuconostoc* spp. have also been investigated as potential probiotics due to their ability to produce antimicrobial peptides and vitamins as well as their capacity to modulate immune responses [7].

Despite numerous studies on *Leuconostoc* taxonomy and evolution, further research on genomic diversity, species dynamics, and functional properties is necessary to fully understand the genus’s potential. The discovery of new *Leuconostoc* species highlights the ongoing exploration of diverse microbial communities in various ecological niches and underscores the importance of continued research to elucidate their characteristics and ecological roles [19,20]. The genus taxonomy has evolved, with the most recent reclassification in 2020 placing *Leuconostoc* within the expanded family of Lactobacillaceae, which now includes the former family Leuconostocaceae [21]. According to the List of Prokaryotic Names with Standing in Nomenclature, the genus *Leuconostoc* currently comprises 17 species [22].

In the past years, the number of available *Leuconostoc* genomes has rapidly increased, recently reaching 553 assemblies. This growth opens the door for a comprehensive phylogenomic analysis of the genus, moving beyond traditional taxonomic methods based on phenotypic and morphological features and chemotaxonomic criteria such as DNA-DNA hybridization, G+C content, and 16S rRNA gene sequencing [23,24]. Comparative genome analysis also allows for detailed comparisons at species or strain levels, giving insights into their functional genetic potential.

This study aimed to uncover the differences in the metabolism of *Leuconostoc* species through a thorough comparative genomic analysis of the genus, leveraging the availability of 453 sequenced genomes. A deep functional annotation was conducted to delineate distinct genotypic traits among the sequenced species and strains, deciphering phenotypic behaviors and unique characteristics. This analysis sheds light on the metabolic potential and ecological adaptations of this group of LAB.

## 2. Materials and Methods

A total of 553 *Leuconostoc* genome sequences were retrieved from NCBI datasets as of 1 October 2023. Only genomes obtained through pure culture sequencing were included, while metagenome-assembled ones were excluded. CheckM [25] was employed to assess genome completeness and contamination.

Genomes were annotated using Prokka [26] for subsequent pangenome calculation with Roary [27], setting the minimum percentage identity parameter at 80%. Functional annotation was performed using eggnog-mapper v2.1.12 [28,29] to assign KEGG numbers to each protein. KEMET [30] was then used to evaluate KEGG module completeness. Presence of Carbohydrate Active enZymes (CAZy) were investigated with the standalone version of the dbCAN3 that incorporates HMMER, Diamond, and dbCAN_sub for annotating CAZyme families [31].

Principal Coordinate Analysis (PCoA) based on the Jaccard dissimilarity index, calculated from the presence/absence of KEGG functions, was conducted using R with the vegan and ape packages [32,33]. The following enzymes for citrate metabolism and the acetoin pathway were searched [5]: citrate permease (AAA60396) of *Leuconostoc mesenteroides* subsp. *mesenteroides*, citrate lyase alpha and beta subunits (CAA71633 and CAA71632) of *L. mesenteroides* subsp. *cremoris*, oxaloacetate decarboxylase (AFS39629) of *Leuconostoc gelidum*, alpha-acetolactate synthase (SPJ44178) of *L. carnosum*, alphaacetolactate decarboxylase (AFT82058) of *L. carnosum*, diacetyl reductase (SPJ42929) of *L. carnosum*, and 2,3-butanediol dehydrogenase (WP_135197409) of *L. carnosum*.

BLASTp searches were also conducted for mannitol dehydrogenase (ACM66886.1) of *L. mesenteroides*, malic enzyme (BAX72645.1) of *L. mesenteroides*, malolactic enzyme (KGB50834.1) of *L. mesenteroides* and the genes responsible for biogenic amine production tyrosine decarboxylase (AAN77279.2) of *Levilactobacillus brevis*, agmatine/putrescine exchanger (ABS19476.1) of *Lv. brevis*, agmatine deiminase (ABS19477.1) of *Lv. brevis*, ornithine decarboxylase (ANJ65946.1) of *Furfurilactobacillus rossiae*, lysine decarboxylase (NP_414728.1) of *Escherichia coli*, and histidine decarboxylase (BAG26233.1) of *Limosilactobacillus reuteri* subsp. *reuteri*.

The Total Average Nucleotide Identity (ANI) was calculated following Gosselin et al. [34] method, with bootstrap values obtained from 100 ANI distance matrix replicates. Alignments of 269 core genes identified by Roary out of a total of 32,467 genes were utilized to generate phylogenetic trees. RAxML tool with 100 bootstrap replicates was used for maximum likelihood tree construction [35]. The ANI distance matrix was employed for tree construction using the script by Gosselin et al. [34]. In this script, the balanced minimum evolution algorithm implemented in the FastME function of the R package ape [33] was applied to generate phylogenies for each distance matrix [36], whereas the function plotBS of the R package Phangorn [37] was exploited to map support values onto the tree. SplitsTree v. 4.18.2 [38] was utilized for additional phylogeny inference, employing a neighbor-net drawing and Jukes–Cantor correction for alignment-derived trees [38,39].

## 3. Results and Discussion

### 3.1. Phylogenomic Organization

A dataset encompassing all 553 genome sequences of *Leuconostoc* species was available on the 1st of October 2023. The sequences of metagenome-assembled genomes, duplicates, and those exhibiting contamination levels exceeding 5% following the CheckM assessment were excluded from the dataset, resulting in a total of 453 genomes that were selected for further analysis (Supplementary Datasheet S1). Most of the genomes were ascribed to all 17 correctly named species (20 *L. carnosum*, 58 *L. citreum*, 13 *L. falkenbergense*, 4 *L. fallax*, 19 *L. gelidum*, 32 *L. gasicomitatum*, 3 *L. holzapfelii*, 1 *L. inhae*, 2 *L. kimchii*, 30 *L. lactis*, 2 *L. litchii*, 1 *L. miyukkimchii*, 219 *L. mesenteroides*, 1 *L. palmae*, 15 *L. pseudomesenteroides*, 1 *L. rapi*, and 23 *L. suionicum*), including the type strains (Supplementary Table S1), while 8 had the general designation *Leuconostoc* sp., and 1 did not have a valid taxonomic name (i.e., *Leuconostoc garlicum*) [22].

Pairwise ANI values were calculated between genomes (Supplementary Datasheet S2). The analysis ascribed each strain to 1 of the 17 recognized species according to an ANI threshold of 95%. The species delineations were consistent with those previously published except for *L. inhae*, which now is separated from *L. gasicomitatum* [40] and identified as G19. *L. gelidum* subsp. *aenigmaticum*, a subspecies included in this work, was correctly clustered with other *L. gelidum* strains. The strains ascribed to *L. lactis* are still split into two phylogenetically related groups, G9 and G10.

**Figure 1:**
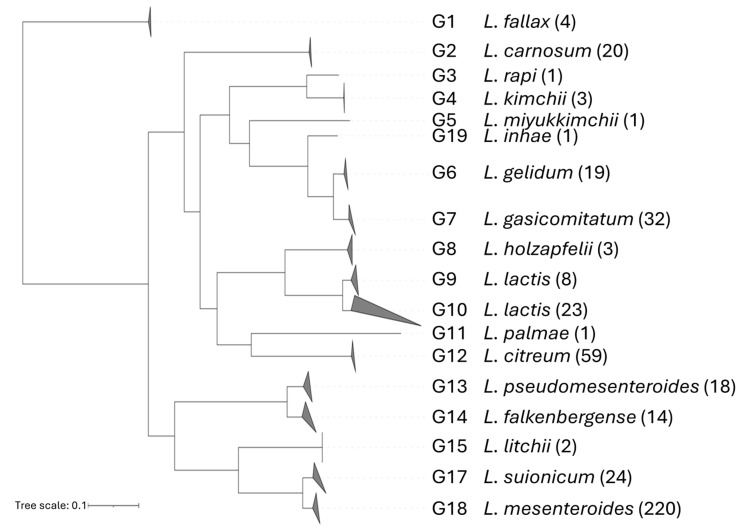
The phylogenomic tree of 453 *Leuconostoc* genomes based on core gene alignment. Genome groups correspond to species as delineated by ANI. For each group, the number of genomes is reported in brackets.

Phylogenetic trees were computed utilizing ANI and core genome alignment. The split decomposition of the trees is consistent with the previous results [40] and confirmed the most recent amendments (Supplementary Figure S1). The phylogenetic relationships between the species are reported in Figure 1.

### 3.2. Functional Analysis

The functional annotation of the 453 genomes carried out with eggnog-mapper allowed us to identify KEGG metabolic blocks for metabolic reconstruction and comparison. A total of 1722 metabolic blocks were identified, 608 shared by all the genomes (Supplementary Datasheet S3). 

**Figure 2:**
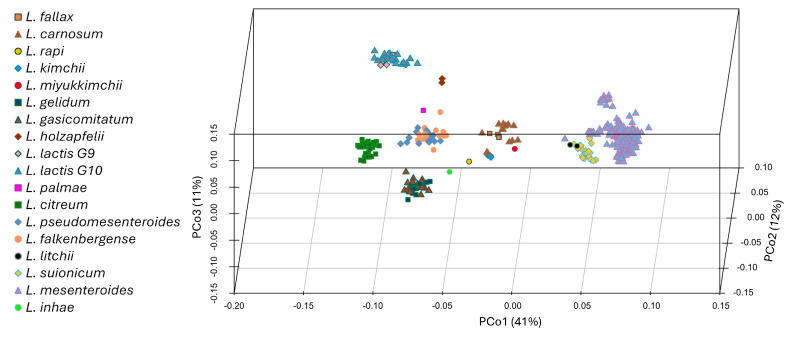
The PCoA based on the Jaccard distance matrix between *Leuconostoc* genomes, computed on the enzymatic functions predicted by KEGG.

A Jaccard distance matrix among strains, then subjected to a PCoA, was computed based on the presence of KEGG metabolic blocks. The species formed well-distinct clusters in the first three dimensions of the PCoA space (Figure 2).

The strains belonging to *L. litchii*, *L. suionicum*, and *L. mesenteroides* were the sole strains laying at positive values of PCo1 (Figure 2). Most of the numerous strains of *L. mesenteroides* were well separated from a cluster encompassing the 23 strains of *L. suionicum* and the 2 *L. litchii*. The strains of the subspecies *L. mesenteroides* subsp. *cremoris* constituted a subgroup, separated along PCo3 from the other *L. mesenteroides*. At negative values of PCo1, the two groups of *L. lactis* (G9 and G10) formed a group at positive PCo2 and PCo3, *L. falkenbergense* and *L. pseudomesenteroides* clustered together at negative PCo2 and positive of PCo3, *C. citreum* was located at positive PCo2 and negative PCo3, and *L. gelidum* and *L. gasicomitatum* clustered at negative PCo2 and slightly negative PCo3. Blocks involved in the biosynthesis of pyridoxal-P, histidine, tryptophan, and sulfur-containing amino acids metabolism were among the major determinants of separation along PCo1. 

### 3.3. Metabolic Reconstruction

**Figure 3:**
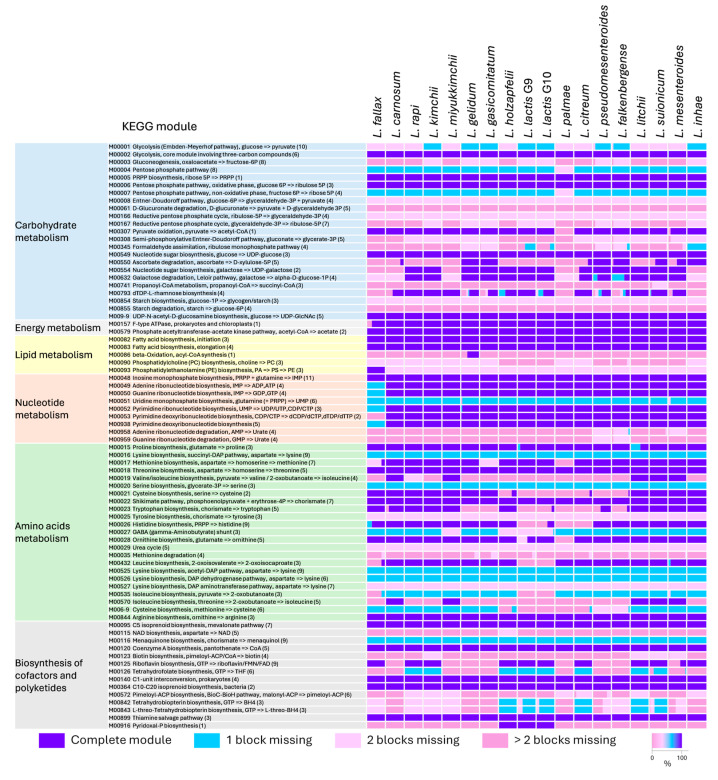
Stacked bars plots reporting the degree of completion of KEGGs metabolic modules in *Leucosnostoc* groups.

**Figure 4:**
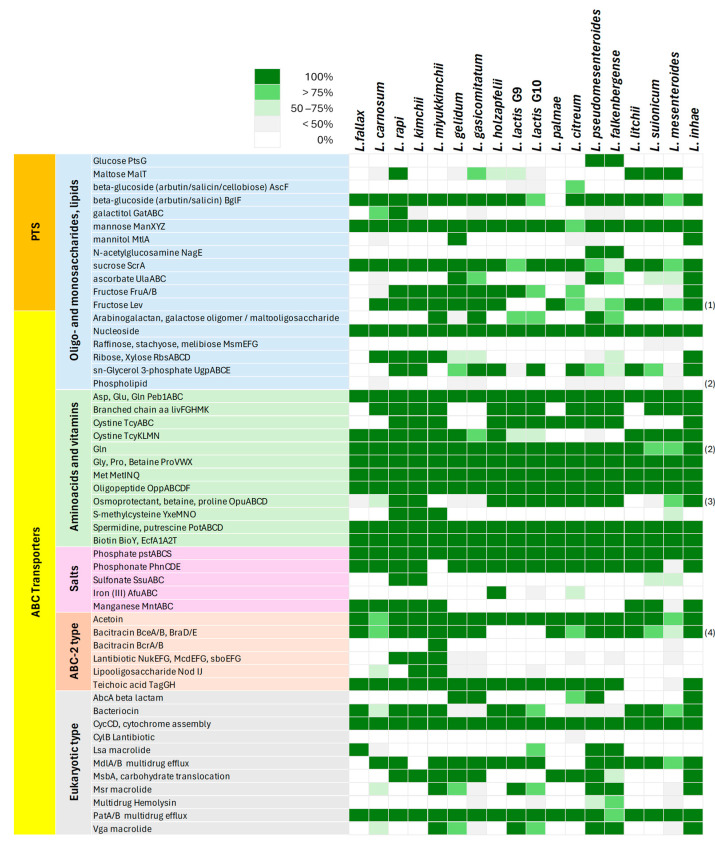
A heatmap of the prevalence of complete PTS and ABC transporters in *Leuconostoc* groups. Shades of green indicate the frequency of the genes in each group. Notes: (1) Only EIID component; (2) Only substrate binding; (3) ATP binding often missing; (4) BraE missing in G2, G3, G4, G5, and G6.

The metabolic potential of *Leuconostoc* bacteria was inferred utilizing information on annotated metabolic functions available in the KEGG database (Supplementary Datasheet S4), coupled, when necessary, with a BLAST search of specific enzymes responsible for specific metabolic blocks and with the search of the enzymes involved in carbohydrate metabolism. The KEGG metabolic modules identified in *Leuconostoc* genomes and their degree of completeness in the different species are summarized in Figure 3, while the prevalence of complete PTS and ABC transporters in the different species is summarized in Figure 4.

#### 3.3.1. Central Catabolic Route

The *Leuconostoc* genus, known for its heterofermentative metabolism, primarily utilizes the pentose phosphate pathway (PPP) and phosphoketolase pathway (PKP) for sugar catabolism, resulting in the production of lactate, acetate, or ethanol and CO_2_. Consistent with its crucial role in the central metabolism, D-xylulose-5-phosphate phosphoketolase (EC 4.1.2.9), the pivotal enzyme in the PKP, is encoded by a gene present in the core genome of all *Leuconostoc* species.

Interestingly, both the Embden–Meyerhof (EMP) and Entner–Doudoroff (ED) pathways, common glycolytic pathways in other organisms, are incomplete in *Leuconostoc* species. The EMP pathway is disrupted at the 6-phosphofructokinase step, and the ED pathway lacks phosphogluconate dehydratase and 2-dehydro-3-deoxy-phosphogluconate aldolase. The alternative pathways identified in leuconostocs allowed for the efficient utilization of the available substrates through less common routes, providing these bacteria with flexibility and adaptation to various environments [41,42].

Moreover, the presence of NADH oxidase across all species indicates a mechanism for maintaining redox balance by regenerating NAD+ through oxygen reduction. This feature might contribute to the survival of these bacteria in oxygen-variable environments, supporting their growth and metabolic activities [42,43].

#### 3.3.2. Uptake and Fermentation of Sugars

Sugar uptake in LAB could take place by primary active transporters, secondary transporters, group translocators, and channels [43,44]. The most common energy coupling mechanisms rely on phosphoenolpyruvate in group translocators, such as phosphotransferase systems (PTS), ATP in primary transporters based on ATB-binding cassettes (ABC), and gradients of cations in symport or antiport secondary transporters [43,44]. Several PTS and ABC transporters were identified through KEGG annotation (Figure 4).

*Leuconostoc* species exhibited a diverse array of phosphotransferase systems (PTS) for sugar uptake, reflecting their adaptability to different sugar substrates. Mannose (Man XYZ), sucrose (ScrA), and beta-glucoside (BglF) PTS transporters were the most widespread, indicating a primary reliance on these sugars. Fructose PTS transporters (FruA/B) were also prevalent in the genomes of the related species *L. rapi*, *L. kimchii*, *L. miyukkimchii*, *L. gelidum*, *L. gasicomitatum*, *L. holzapfelii*, *L. lactis*, *L. citreum*, and *L. inhae*, underscoring fructose’s significance in their metabolism. 

Other PTS transporters, such as those for glucose (PtsG) and N-acetylglucosamine (NagE), were restricted to specific species like *L. pseudomesenteroides* and *L. falkenbergense*, suggesting niche adaptations. Similarly, maltose PTS transporters (MalT) were found in all the genomes of *L. rapi*, *L. litchii*, *L. suionicum*, and *L. mesenteroides* and most genomes of *L. gasicomitatum*, *L. holzapfelii*, and *L. lactis* (G9), suggesting a role in environments where maltose is present [45].

Galactitol PTS (GatABC) distinguished *L. rapi* and *L. carnosum*, while ascorbate PTS (UlaABC) characterized most genomes of *L. gelidum*, *L. gasicomitatum*, *L. pseudomesenteroides*, *L. falkenbergense*, *L. suionicum*, *L. mesenteroides*, and *L. inhae*.

A limited number of ABC transporters for the uptake of carbohydrates were identified. A transporter for pentoses (RbsABCD) was found in all the genomes of *L. carnosum*, *L. rapi*, *L. kimchii*, *L. miyukkimchii*, and *L. inhae* and in the majority of *L. gelidum*, *L. gasicomitatum*, and *L. falkenbergense*. A transporter for malto- and galactooligosaccharides characterized most or all *L. miyukkimchii*, *L. gasicomitatum*, *L. lactis*, *L. pseudomesenteroides*, and *L. falkenbergense*.

Unlike PTS and ABC transporters, the secondary transporters relying on the symport or the antiport of cations are not annotated in the KEGG orthology database, but several sequences could be found with an NCBI annotation. In particular, many members of the major facilitator superfamily (MFS) were found in all the genomes of *Leuconostoc* type strains, in numbers ranging from 17 of *L. palmae* to 41 of *L. gelidum* (Supplementary Table S2). Unfortunately, NCBI annotation did not allow for any definition of substrate specificity that could include simple sugars, oligosaccharides, metabolites, amino acids, oxyanions, and xenobiotics [46]. Thus, it is very plausible that they function in the uptake of carbohydrates in *Leuconostoc* also. 

**Figure 5:**
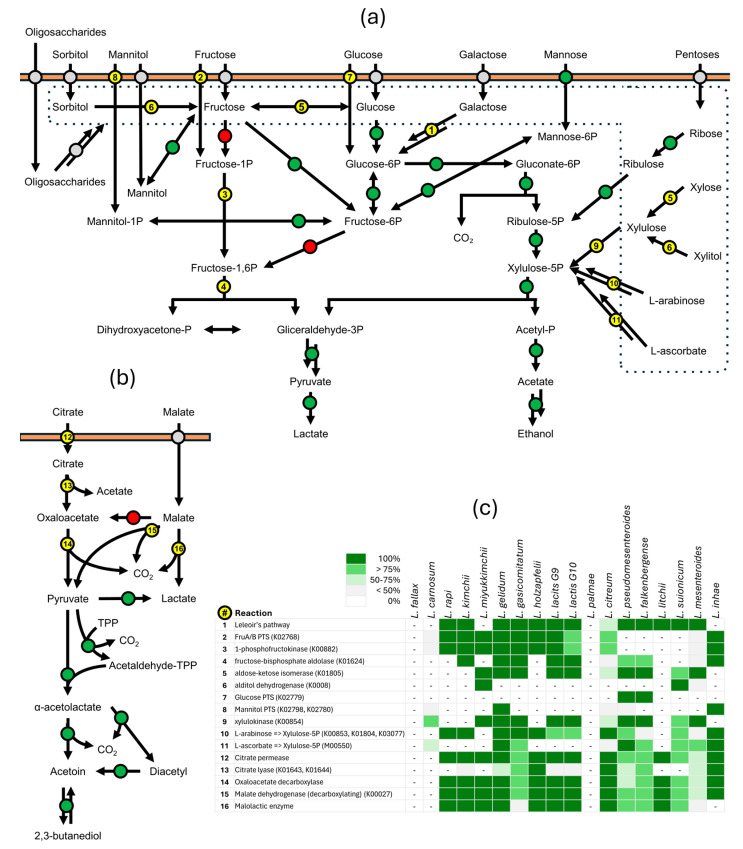
Fermentative pathways predicted from *Leuconostoc* genomes. (**a**) Fermentation of simple sugars. (**b**) Citrate and malolactic fermentations. Colors: green, always present; red, always absent; yellow, not generally present (refer to panel c); grey, not conclusively characterized. (**c**) Occurrence and prevalence of specific metabolic reactions in *Leuconostoc* species.

The uptake mechanisms participated in various specific pathways for sugar catabolism, as elucidated in Figure 3 and Figure 5. For instance, intracellular fructose that could have entered the cell by means of an ABC transporter or could have been generated by intracellular hydrolysis of fructans can be processed in all species via fructose-6P by fructokinase (K00847), then isomerized to glucose-6P by glucose-6-phosphate isomerase (K01810) and channeled towards the heterolactic pathway. Alternatively, in the species *L. kimchii*, *L. gelidum*, *L. gasicomitatum*, *L. lactis* (G9 and G10), and *L. inhae*, fructose could enter the cells as fructose-1P by means of FruA/B PTS (K02768), then be transformed into fructose-1,6 bisphosphate by 1-phosphofructokinase (K00882) and channeled towards the homolactic route by fructose-bisphosphate aldolase (K01624). This dual capability for fructose catabolism highlights a metabolic versatility that likely provides an ecological advantage.

Otherwise, this second route seems precluded in *L. fallax*, *L. carnosum*, *L. palmae*, *L. litchii*, *L. suionicum*, and *L. mesenteroides* that lack FruA/B PTS, 1-phosphofructokinase, and fructose-bisphosphate aldolase. *L. pseudomesenteroides* and *L. falkenbergense* lacked FruA/B PTS and 1-phosphofructokinase, while *L. citreum*, *L. rapi*, *L. miyukkimchii*, and *L. holzapfelii* lacked the aldolase. All the genomes of *L. miyukkimchii*, *L. gelidum*, *L. gasicomitatum*, *L. lactis* (G9 and G10), *L. pseudomesenteroides*, *L. falkenbergense*, *L. suionicum*, *L. mesenteroides*, and some *L. citreum* harbored a gene encoding aldose-ketose isomerase (K01805), which could isomerize glucose into fructose and vice versa.

Leloir’s pathway for galactose utilization was complete in all the species except in *L. fallax*, *L. carnosum*, *L. miyukkimchii*, *L. palmae*, *L. inhae*, and some strains of *L. citreum*, *L. pseudomesenteroides*, and *L. falkenbergense*. In general, the missing genes were those encoding galactokinase (EC 2.7.1.6) and hexose-1-phosphate uridylyltransferase (2.7.7.12).

The presence of enzymes like mannitol dehydrogenase and aldose-ketose isomerase further underscored the metabolic flexibility of *Leuconostoc* species [43]. These enzymes enable the interconversion of sugars like fructose and mannitol and glucose and fructose, respectively, allowing for the bacteria to efficiently exploit a wide range of substrates. *L. miyukkimchii* and *L. suionicum* possessed the gene encoding sorbitol dehydrogenase (K0008) to convert sorbitol into fructose. All the strains were capable of importing mannose through mannose PTS (K02793) and isomerizing it into fructose 6-P with mannose-6-phosphate isomerase (K01809).

*Leuconostoc* species present mechanisms for pentose metabolism, crucial for their growth on plant-derived substrates rich in these sugars [43]. Pentoses can be channeled in the PKP pathway at the level of the intermediates ribulose 5P or xylulose 5P. Genes encoding enzymes for the conversion of ribose into ribulose-5P are ubiquitous, facilitating the entry of ribose into the PKP pathway. However, the inability to convert D-arabinose into ribulose 5P in all species suggests selective pressure to specialize in certain sugars over others.

For xylose metabolism, in the species *L. miyukkimchii*, *L. gelidum*, *L. gasicomitatum*, *L. lactis* (G9 and G10), *L. pseudomesenteroides*, *L. falkenbergense*, *L. suionicum*, and *L. mesenteroide*, the presence of xylulokinase (K00854), transforming xylulose into xylulose 5P, and aldose-ketose isomerase, for the xylose transformation into xylulose, indicates an efficient conversion pathway of xylose to xylulose 5P, integrating into the PKP pathway. Additionally, the presence of xylitol dehydrogenase in the species *L. miyukkimchii* and *L. suionicum* points to an alternative pathway for xylose catabolism, providing a metabolic redundancy that might enhance survival under varying conditions.

Enzymes for the utilization of L-arabinose through transformation into L-ribulose, followed by phosphorylation into L-ribulose 5P and conversion into D-xylulose 5P, were present in *L. rapi*, *L. kimchi*, *L. inhae*, *L. gelidum*, *L. gasicomitatum*, *L. holzapfelii*, *L. palmae*, *L. citreum*, *L. suionicum*, and some strains of *L. lactis* (G9 and G10) and *L. mesenteroides*. 

For simple sugars, reconstructed metabolic capabilities were in good agreement with fermentative profiles of type strains obtained with API 50CH tests (Supplementary Datasheet S5), available as phenotypical information in BacDive (https://bacdive.dsmz.de/api-test-finder). The few discordances may be attributed to the uncertainty of the prediction of uptake systems.

#### 3.3.3. Carbohydrate-Active Enzymes (CAZymes)

Carbohydrate-active enzymes (CAZymes) are families of enzymes that catalyze the degradation, modification, or synthesis of complex carbohydrates. CAZymes play a vital role in the breakdown and fermentation of carbohydrates and in the synthesis of bacterial components such as the peptidoglycan and exopolysaccharides (EPS). Thus, the array of CAZymes harbored by *Leuconostoc* spp. directly impact the ability of these bacteria to thrive in environments rich in complex carbohydrates and have a direct effect on microbial ecology and the evolution (e.g., the ripening vs. the deterioration) of the food matrixes where *Leuconostoc* members are naturally present. Like other LAB, *Leuconostoc* spp. are known producers of EPS. These molecules impact the texture of food matrixes, where they influence viscosity, syneresis, firmness, and the technological and sensory properties [47].

**Figure 6:**
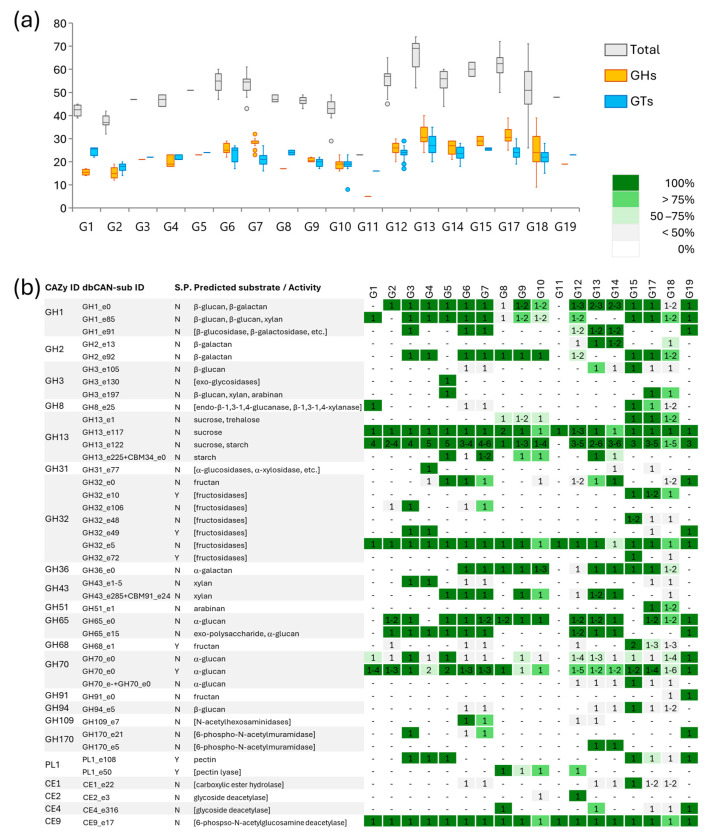
CAZymes predicted in *Leuconostoc* genomes. (**a**) The abundance of total CAZymes, glycosyl hydrolases (GHs), and glycosyl transferases (GTs) in phylogenomic groups; boxes depict the 25th, 50th, and 75th percentiles, whiskers. (**b**) The heatmap of GH and PL presence in *Leuconostoc* groups. Only the GHs and the PLs occurring in at least one group with 100% prevalence and not involved in peptidoglycan processing (i.e., GH23, GH25, and GH73) are reported. S.P. column reports the presence (Y) or absence (N) of the signal peptide. The substrate specificity of each dbCAN-sub is given if available, otherwise generic activity deducted form CAZy ID is given in brackets. The number (or the range) of genes is given in the cells.

Genes encoding a total of 305 different carbohydrate-active enzymes were identified in *Leuconostoc* genomes, 57 of which harbored a signal peptide putatively enabling secretion (Supplementary Datasheet S5). The CAZymes included 126 glycosyl hydrolases (GHs), 146 glycosyl transferases (GTs), 7 carbohydrate esterases (CEs), and 6 polysaccharide lyases (PLs). The number of CAZyme genes harbored by each genome ranged from 23 to 77, most of the genes occurring in a single copy. Remarkable exceptions were GH13_e122, GH70_e0, and GT2, respectively, occurring with up to 6, 6, and 15 copies. The number of GHs and GTs lay in the range of 5–40 and 8–37 per genome, with marked differences among groups (Figure 6). These enzymes enable *Leuconostoc* species to thrive in environments rich in complex carbohydrates, such as plant material and fermented foods [48].

For instance, *L. pseudomesenteroides* was the species harboring among the highest number of GHs and GTs (24–40 and 20–35, respectively), while *L. carnosum* presented among the lowest ones (12–19 and 14–20, respectively). The range was remarkably broad within *L. mesenteroides*, especially due to *L. mesenteroides* subsp. *cremoris*, which uniformly harbored very low numbers of both GH (generally < 15) and GT (generally < 18) genes, unlike other *L. mesenteroides* strains. Nineteen members of GH73 and thirteen of GH25, endo-β-1,4-N-acetylglucosaminidases and lysozyme-like 1,4-N-acetylmuramidases, respectively, both involved in peptidoglycan processing, were widespread across all the groups. Both GH73 and GH25 and, in some cases, specific members of these families (i.e., GH73_e248 and GH73_e84) were found in all genomes, highlighting their role in cell wall remodeling and possibly defense mechanisms against microbial competition [49]. Thirteen members of the GH13 family, with predicted substrate specificity for sucrose and/or starch, were identified, mainly lacking the signal peptide. Some of them occurred in all the genomes of certain groups but were absent in others, while others (e.g., GH13_e117 and GH13_e122) were almost ubiquitous and occurred also in multiple copies. GH32 CAZYmes, encompassing sucrose, fructan, and inulin hydrolases, were found in all the genomes. Of the 13 members of GH32, the intracellular GH32_e5 was found in most of the genomes, while others specifically occurred in some species. Extracellular GH32 were much less widespread, with GH32_e10 characterizing *L. litchii*, *L. suionicum*, and some of *L. mesenteroides* genomes and GH32_e49 occurring only in *L. rapi*, *L. kimchii*, and *L. inhae*. Another extracellular fructosidase belonging to GH68 was predicted in *L. litchii* and in many genomes of *L. suionicum* and *L. mesenteroides*, while an intracellular one belonging to GH91 characterized *L. inhae*. The invertases/fructanases of families GH13 and GH32 were the sole oligosaccharides hydrolyzing GHs found in the species *L. palmae*, which was the least provided of *Leuconostoc* genus.

Intracellular GH1 and GH2 members with predicted specificity for β-glucosides and/or β-galactosides were identified in most species, in some of them in multiple copies. Most or all the genomes of *L. rapi*, *L. kimchii*, *L. miyukkimchii*, *L. gelidum*, *L. gasicomitatum*, *L. lactis* (G9 and G10), *L. citreum*, *L. litchii*, *L. suionicum*, and *L. inhae* harbored both GH1_e0 and GH1_e85, while *L. carnosum* harbored only the former and *L. fallax* only the latter. Most of the genomes of *L. mesenteroides* harbored both the sequences, or at least GH1_e85, with the exception of *L. mesenteroides* subsp. *cremoris* and some other strains that lacked both. GH2 members (GH2_e13 and GH2_e92) were identified in all or most strains of *L. rapi*, *L. kimchii*, *L. gelidum*, *L. gasicomitatum*, *L. holzapfelii*, *L. lactis* (G9 and G10), *L. citreum*, *L. pseudomesenteroides*, *L. falkenbergense*, *L. litchii*, *L. suionicum*, and *L. mesenteroides*. The presence of GH1 and GH2 families further supports the ability of *Leuconostoc* species to utilize diverse carbohydrates, including those derived from plant cell walls [50].

Two GH43 1,4-β-xylosidases were identified, one characterizing the genomes *L. rapi* and *L. kimchii* and the other *L. miyukkimchii*, *L. gelidum*, *L. gasicomitatum*, *L. lactis*, *L. pseudomesenteroides*, and *L. falkenbergense.* Intracellular GH65 and GH70 members with predicted specificity for alpha-glucosides were identified in most species, in some of them in multiple copies. The sequences of an extracellular member of GH70 were also widespread, being highly prevalent in most species. An intracellular alpha-galactosidase belonging to GH36 was found in all the genomes of *L. gelidum*, *L. gasicomitatum*, *L. holzapfelii*, *L. lactis*, *L. pseudomesenteroides*, *L. falkenbergense*, *L. litchii*, and *L. suionicum* and in most of *L. mesenteroides*.

Other intracellular CAZymes belonging to GH3, GH8, GH31, GH51 GH94, GH109, and GH170, responsible for the hydrolysis of a variety of glycans (e.g., alpha and beta glucans and hexosamines-containing polysaccharides), were less frequent and characterized the genome of certain species.

Among the glycosyl transferases, GT2, GT4_e1712, GT4_e199, GT4_e2671, GT4_e272, GT28_e1, GT51_e10, GT51_e50, GT111_e1 (or in alternative GT111_e12), and GT113_e35 were found in the vast majority of the genomes in all the species, with only a few exceptions (Supplementary Figure S2). These GTs are active on UDP- or GDP-activated hexoses and involved in the biosynthesis of glycans, including peptidoglycan and EPS. The high abundance of GT2 sequences, with up to 15 copies per genome (while all the others generally occurred with one or, more rarely, two copies), underscores their crucial role in carbohydrate metabolism and in the stability of bacterial cells [51]. Other GTs were diversely distributed in the genus, in some cases presenting a remarkably high prevalence in one or a few species. Such diversity in the array of GTs is expected to affect the biosynthetic properties of oligo- and polysaccharides and could thus influence the structure of the EPS. However, information on the substrate specificity of the GTs cannot be inferred with precision from the CAZy database. As a matter of fact, *Leuconostoc* is one of the major EPS producers among LABs, and increasing structural information has been recently obtained from specific strains of *L. mesenteroides*, *L. pseudomesenteroides*, and *L. citreum* [52,53,54]. The EPS of *L. pseudomesenteroides* was mostly a linear α-(1→6)-linked glucan [52] that also contained mannose residues in *L. mesenteroides* [53], whereas it was a highly branched structure rich in uronic acids in *L. citreum* [54]. A thorough structural characterization and comparison of the EPS among *Leuconostoc* species still needs to be carried out to establish some correspondence with the GTs harbored in the genomes.

Of the six PL, two PL1 sequences with putative pectin lyase activity were found with high prevalence in some species, i.e., the extracellular PL1_e108 and PL1_e50. The former was found in all or most of the genomes of *L. rapi*, *L. kimchii*, *L. miyukkimchii*, *L. litchii*, *L. suionicum*, and *L. inhae*, while the latter in *L. holzapfelii*, *L. lactis* (G9 and G10), and *L. citreum*.

Among esterase, a sequence of CE9_e17a putative N-acetylglucosamine 6-phosphate deacetylase was found in nearly all the genomes. Other CEs characterized a more limited number of genomes, such as the glycoside deacetylase CE4_31 characterizing most *L. gelidum*, *L. gasicomitatum*, *L. holzapfelii*, *L. pseudomesenteroides*, and *L. inhae*; the glycoside deacetylase CE2_13 in all the genomes of *L. citreum*; and the esterase CE1 in *L. litchii.*

Unlike for simple sugars, it was not possible to establish a relationship between predicted CAZymes and the observed API 50CH phenotypes, with regards to the fermentation of oligo- and polysaccharides (e.g., inulin, raffinose, maltose), mainly due to the lack of sufficient information regarding the substrate specificity of GHs and the other CAZymes (Supplementary Datasheet S6). For instance, fructosidases and alpha-glucanases are very widespread, but they likely targeted only di- and oligosaccharides, while no strains could utilize inulin and starch.

#### 3.3.4. Metabolism of Organic Acids

Organic acids such as citrate and malate are metabolized by some *Leuconostoc* species, impacting the sensory properties of dairy products and wine, respectively [43,55]. Citrate is present in fruit juices, milk, and vegetables and is also added as a preservative in foods. The complex metabolism of citrate can generate 4-carbon compounds, such as diacetyl, acetoin, and butanediol, which possess aromatic and slight antimicrobial properties [43,56]. These compounds play a pivotal role in the fermentation processes involving certain *Leuconostoc* species. The ability to metabolize citrate and other organic acids contributes significantly to the flavor development and sensory properties of dairy products and other fermented foods [57,58]. Additionally, this metabolic capability aids in the preservation of fermented foods. The enzymes/blocks involved in citrate uptake and transformation into pyruvate (i.e., citrate permease, α and β citrate lyase, and oxalacetate decarboxylase) were predicted in the species *L. holzapfelii*, *L. citreum*, and *L. inhae* and in several strains belonging to *L. gasicomitatum*, *L. gelidum*, *L. pseudomesenteroides*, *L. falkenbergense*, and *L. mesenteroides* (including all the *L. mesenteroides* subsp. *cremoris*). In general, in the other species and in the remaining strains of *L. gasicomitatum*, *L. gelidum*, *L. pseudomesenteroides*, *L. falkenbergense*, and *L. mesenteroides*, the citrate pathway was absent (e.g., in *L. fallax* and *L. carnosum*) or lacked only the subunits of citrate lyase (e.g., *L. lactis* and *L. suionicum*) (Figure 5).

Citrate metabolism not only provides an additional energy source but also contributes to the production of key flavor compounds like diacetyl and acetoin. The genes encoding the enzymes involved in the transformation of pyruvate into acetoin, diacetyl, and 2,3-butanediol (i.e., α-acetolactate synthase, α-acetolactate decarboxylases, diacetyl acetoin reductases, and 2,3 butanediol dehydrogenase) belonged to the core genome of the genus.

Malolactic conversion, also referred to as malolactic fermentation, consists of the decarboxylation of malic acid, catalyzed by malolactic enzyme, to yield lactic acid. Such a reaction, which converts malate to lactate, is carried out by an array of LAB, including some *Leuconostoc* members, and represents an important metabolic pathway, crucial for deacidifying fermented products, enhancing their sensory properties and improving microbial stability [59]. In particular it is relevant in winemaking, where malic acid that naturally occurs in grape must is converted to softer-tasting lactic acid.

The malolactic enzyme was predicted in all the genomes of *L. rapi*, *L. kimchii*, *L. miyukkimchii*, *L. gelidum*, *L. holzapfelii*, *L. lactis* (G9 and G10), *L. citreum*, and *L. litchii*; in most of *L. pseudomesenteroides*, *L. falkenbergense*, *L. suionicum*; and a minority of *L. gasicomitatum* and *L. mesenteroides* (Figure 5). The reaction could also take place by being catalyzed by decarboxylating malate dehydrogenases [60]. Interestingly, the same sequence retrieved in *Leuconostoc* genomes received the annotation of both this latter activity and oxalacetate decarboxylase. The widespread presence of this pathway indicates its ecological relevance and has significant impacts on the industrial applications of these microorganisms. Malate dehydrogenase, yielding oxaloacetate, was absent in all the genomes.

The degradation of ascorbate was assessed in some species of lactobacilli, which could lead to a decrease in this preservative, thus contributing to food deterioration [61]. The route of ascorbate degradation consists of lactone opening, tautomerization, and decarboxylation to yield xylulose 5-phosphate, which is catabolized in the pentose phosphate pathway. The route was complete in all the strains of *L. gelidum* subsp. *gelidum* and *L. gelidum* subsp. *aenigmaticum*, *L. gasicomitatum*, *L. pseudomesenteroides*, *L. falkenbergense*, and *L. inhae* and in some strains of *L. carnosum*, *L. citreum*, *L. suionicum*, and *L. mesenteroides*. In all the other species and in the remaining strains of *L. carnosum*, *L. citreum*, *L. suionicum*, and *L. mesenteroides*, the ascorbate degradation pathway was absent. Information on ascorbate degradation in food by leuconostocs is still lacking. A possible role of leuconostocs in ascorbate degradation deserves to be investigated in food matrixes to establish whether they could contribute to food deterioration.

#### 3.3.5. Metabolism of Amino Acids and Cofactors

ABC transporters for the intake of many preformed aspartate, glutamate, glutamine, glycine, proline, cystine (TcyABC or TcyKLMN), and methionine peptides; for biogenic amines; and for peptides were ubiquitously found in all the *Leuconostoc* genomes and indicate a high dependence on exogenous amino acids (Figure 4). An ABC transporter for the intake of branched chain amino acids was also very widespread, characterizing all the genomes except those of *L. fallax*, *L. gelidum*, *L. gasicomitatum*, *L. palmae*, and *L. litchii*. A betaine/proline transporter was also found in most species, while the one for S-methylcysteine characterized only *L. rapi*, *L. kimchii*, and *L. miyukkimchii*. *Leuconostoc* species exhibit both the capacity to efficiently import essential nutrients from their surroundings and a complex and varied ability to metabolize amino acids, highlighting their adaptation to diverse ecological niches and nutritional environments. 

The genes encoding glutamate dehydrogenase and alanine dehydrogenase were missing in all the genomes, indicating that ammonium organication into these amino acids is precluded. On the other hand, glutamic-aspartic transaminase was ubiquitous, and glutamate synthase was predicted only in all the genomes of *L. suionicum* and in a minority of *L. mesenteroides*. The pathway of serine production from glycolysis intermediates seemed ineffective due to the lack of phosphoserine phosphatase in all the genomes, indicating a reliance on external sources of serine consistent with the organism’s adaptation to environments rich in preformed amino acids.

The pathways depending on preformed serine to yield glycine and cysteine; on glutamate to yield glutamine, arginine, and proline; and on aspartate to yield homoserine and threonine were generally complete (Figure 3). Asparagine could be produced from aspartate by aspartate ammonia lyase (K01914) in 66 genomes, mainly belonging to *L. mesenteroides*. Otherwise, asparagine production could be achieved from oxalacetate by omega-amidase and asparagine-keto acid aminotransferase (K13566 and K22457) in all the species except *L. fallax*. The pathway from homoserine to methionine was always complete except in *L. palmae*, *L. gasicomitatum*, and *L. fallax*. 

The pathway of lysine biosynthesis was complete from aspartate to 2,3,4,5-tetrahydrodipicolinate. Succinylase, acetylase, and dehydrogenase pathways required to convert 2,3,4,5-tetrahydrodipicolinate into lysine were incomplete, missing one or two enzymes. Notably, the carboxy-lyase enzyme responsible for forming lysine from meso-2,6-diaminoheptanedioate, the common intermediate of the three pathways, was always present.

The anabolic pathways leading to branched chain amino acids were complete only in *L. carnosum*, *L. miyukkimchii*, *L. litchii*, *L. suionicum*, and *L. mesenteroides*.

With regard to aromatic amino acids, the shikimate pathway from erythrose 4-phosphate to chorismate was complete. The pathways leading to tyrosine and phenylalanine both seemed interrupted due to the lack of chorismate mutase and also always lacked other enzymes. Tryptophan biosynthesis was complete in *L. fallax*, *L. rapi*, *L. kimchii*, *L. miyukkimchii*, *L. holzapfelii*, *L. litchii*, *L. suionicum*, *L. mesenteroides*, and *L. inhae*; in most of *L. carnosum* and *L. pseudomesenteroides*; and in a minority of *L. lactis* and *L. falkenbergense*. 

The route from 5-phosphoribosyl diphosphate to histidine was complete except for *L. lactis*, *L. palmae*, and *L. citreum*.

The genes encoding histidine, lysine, tyrosine, and ornithine decarboxylases (hdcA, ldc, tyrDC, and odc, respectively) and agmatine deiminase (aguD and aguA), responsible for the synthesis of biogenic amines, were absent in all the genomes. 

With regards to vitamins, the thiamine salvage pathway is ubiquitous in all the genomes (Figure 3). Pyridoxal phosphate biosynthesis characterized only *L. holzapfelii* and *L. lactis* (G9 and G10) but was lacking in all the other species. Riboflavin biosynthesis is present in a subset of species (*L. fallax*, *L. rapi*, *L. kimchii*, *L. holzapfelii* and *L. lactis* (G9 and G10), *L. citreum*, *L. litchii*, *L. suionicum*, *L. inhae*, and some *L. mesenteroides)*, indicating varying capabilities to synthesize this essential vitamin. Similarly, tetrahydrofolate biosynthesis, crucial for nucleotide synthesis and amino acid metabolism, is found (lacking alkaline phosphatase) only in *L. rapi*, *L. kimchii*, *L. holzapfelii* and *L. lactis* (G9 and G10), *L. citreum*, *L. litchii*, and most *L. suionicum*, suggesting a selective evolutionary adaptation. 

The pathway of mevalonate production and the subsequent C10–C20 isopreonid biosynthesis towards geranyl geranyl-PP, critical for synthesizing vital cellular components, such as quinones, were complete in all the genomes (Figure 3). In all genomes, menaquinone biosynthesis from chorismate to menaquinol lacked only 1,4-dihydroxy-2-naphthoyl-CoA hydrolase. 

## 4. Conclusions

With the advent of the genomic era, the increasing availability of bacterial genomes has significantly enhanced our understanding of bacterial taxa. Recent comprehensive studies on the genus *Leuconostoc* [41,49] have primarily focused on species delineation, refining the taxonomy of certain *Leuconostoc* groups and resolving issues such as paraphyletic branches. However, a detailed investigation into the functions and metabolic potential of this genus has not been thoroughly conducted until now.

In this study, we elucidated the metabolic potential of the species belonging to this genus. Their metabolic pathways exhibit remarkable adaptability to diverse sugar substrates through specialized transporters and catabolic enzymes. The ability of these species to import and utilize a wide range of amino acids and vitamins from their environment along with their selective biosynthetic pathways enables them to thrive in various competitive habitats. This metabolic diversity not only supports their role in natural ecosystems but also underscores their potential in industrial applications. In particular, their capability to metabolize a wide range of substrates and affect sensory properties and texture is invaluable in the fermentation industry, where they can be utilized to produce various fermented foods and bioproducts. On the other hand, these same properties could be undesired in certain food or beverages, where leuconostocs could participate to spoilage and deterioration. The ongoing exploration of their genomic and metabolic capabilities continues to unveil the versatility and potential of the *Leuconostoc* genus.

## Data Availability

GenBank accession numbers of the genomes are reported in Supplementary Datasheet S1. The original contributions presented in the study are included in the article and supplementary materials. Further inquiries can be directed to the corresponding author.

## Supplementary Materials

The supporting information can be downloaded at https://www.mdpi.com/article/10.3390/microorganisms12071487/s1, Supplementary Table S1: Nominal taxonomy of the analyzed genomes; Supplementary Table S2: Number of transporters belonging to Major Facilitator Superfamily (MFS), as annotated by NCBI in the genome of *Leuconostoc* type-strains; Supplementary Figure S1. Split decomposed phylogenetic trees based on (a) core genes alignment and (b) ANI values; Supplementary Figure S2: Heatmap of GTs presence in *Leuconostoc* groups. S.P. column reports the presence (Y) or absence (N) of the signal peptide. The number (or the range) of genes is given in the cells; Supplementary Datasheet S1: List of *Leuconostoc* genomes analyzed in the present study; Supplementary Datasheet S2: Matrix of pairwise ANI between 453 *Leuconostoc* genomes; Supplementary Datasheet S3: KEGG functions predicted in *Leuconostoc* genomes; Supplementary Datasheet S4: Degree of Completness of KEGG modules in *Leuconostoc* genomes; Supplementary datasheet S5: Fermentative profiles of type strains obtained using the API 50CH test, as reported in the BacDive database. For more information, please visit the BacDive API Test Finder at https://bacdive.dsmz.de/api-test-finder; Supplementary Datasheet S6: CAZymes predicted in *Leuconostoc* genomes utilizing dbCAN3.

## References

[1] Nieminen T.T., Säde E., Endo A., Johansson P., Björkroth J., Rosenberg E., DeLong E.F., Lory S., Stackebrandt E., Thompson F. (2014). The Family *Leuconostocaceae*. The Prokaryotes.

[2] Bello S., Rudra B., Gupta R.S. (2022). Phylogenomic and comparative genomic analyses of *Leuconostocaceae* species: Identification of molecular signatures specific for the genera *Leuconostoc*, *Fructobacillus* and *Oenococcus* and proposal for a novel genus *Periweissella* gen. nov. Int. J. Syst. Evol. Microbiol..

[3] Dellaglio F., Dicks L.M.T., Torriani S., Wood B.J.B., Holzapfel W.H. (1995). The Genus *Leuconostoc*. The Genera of Lactic Acid Bacteria.

[4] Yu A.O., Leveau J.H.J., Marco M.L. (2020). Abundance, diversity and plant-specific adaptations of plant-associated lactic acid bacteria. Environ. Microbiol. Rep..

[5] Candeliere F., Raimondi S., Spampinato G., Tay M.Y.F., Amaretti A., Schlundt J., Rossi M. (2021). Comparative Genomics of *Leuconostoc carnosum*. Front. Microbiol..

[6] Vedamuthu E.R. (1994). The dairy *Leuconostoc*: Use in dairy products. J. Dairy Sci..

[7] Raimondi S., Spampinato G., Candeliere F., Amaretti A., Brun P., Castagliuolo I., Rossi M. (2021). Phenotypic Traits and Immunomodulatory Properties of *Leuconostoc carnosum* Isolated From Meat Products. Front. Microbiol..

[8] Alegria A., Delgado S., Florez A.B., Mayo B. (2013). Identification, typing, and functional characterization of *Leuconostoc* spp. strains from traditional, starter-free cheeses. Dairy Sci. Technol..

[9] Samet-Bali O., Bellila A., Ayadi M.-A., Marzouk B., Attia H. (2020). A comparison of the physicochemical, microbiological and aromatic composition of Traditional and Industrial Leben in Tunisia. Int. J. Dairy Technol..

[10] Tsitko I., Manninen J., Smart K., James S., Laitila A. (2018). Management of barley-associated bacterial biofilms: A key to improving wort separation. J. Inst. Brew..

[11] Shin S.Y., Han N.S., Liong M.T. (2015). Leuconostoc spp. as starters and their beneficial roles in fermented foods. Beneficial Microorganisms in Food and Nutraceuticals. Microbiology Monographs.

[12] Venegas-Ortega M.G., Flores-Gallegos A.C., Martínez-Hernández J.L., Aguilar C.N., Nevárez-Moorillón G.V. (2019). Production of bioactive peptides from lactic acid bacteria: A sustainable approach for healthier foods. Compr. Rev. Food Sci. Food Saf..

[13] Ahmadi-Ashtiani H.-R., Baldisserotto A., Cesa E., Manfredini S., Sedghi Zadeh H., Ghafori Gorab M., Khanahmadi M., Zakizadeh S., Buso P., Vertuani S. (2020). Microbial Biosurfactants as Key Multifunctional Ingredients for Sustainable Cosmetics. Cosmetics.

[14] Costa S., Summa D., Semeraro B., Zappaterra F., Rugiero I., Tamburini E. (2021). Fermentation as a strategy for bio-transforming waste into resources: Lactic acid production from agri-food residues. Fermentation.

[15] Ogier J.C., Casalta E., Farrokh C., Saihi A. (2008). Safety assessment of dairy microorganisms: The *Leuconostoc* genus. Int. J. Food Microbiol..

[16] Ghobrial M., Ibrahim M., Streit S.G., Staiano P.P., Seeram V. (2023). A Rare Case of *Leuconostoc pseudomesenteroides* Bacteremia and Refractory Septic Shock. Cureus.

[17] Modaweb A., Mansoor Z., Alsarhan A., Abuhammour W. (2022). A Case of Successfully Treated Central Line-Associated Bloodstream Infection Due to Vancomycin-Resistant *Leuconostoc citreum* in a Child With Biliary Atresia. Cureus.

[18] Hosoya S., Kutsuna S., Shiojiri D., Tamura S., Isaka E., Wakimoto Y., Nomoto H., Ohmagari N. (2020). *Leuconostoc lactis* and *Staphylococcus nepalensis* Bacteremia, Japan. Emerg. Infect. Dis..

[19] Antunes A., Rainey F.A., Nobre M.F., Schumann P., Ferreira A.M., Ramos A., Santos H., da Costa M.S. (2002). *Leuconostoc ficulneum* sp. nov.; a novel lactic acid bacterium isolated from a ripe fig, and reclassification of *Lactobacillus fructosus* as *Leuconostoc fructosum* comb. nov. Int. J. Syst. Evol. Microbiol..

[20] Leisner J.J., Vancanneyt M., Van der Meulen R., Lefebvre K., Engelbeen K., Hoste B., Laursen B.G., Bay L., Rusul G., De Vuyst L. (2005). *Leuconostoc durionis* sp. nov.; a heterofermenter with no detectable gas production from glucose. Int. J. Syst. Evol. Microbiol..

[21] Zheng J., Wittouck S., Salvetti E., Franz C.M.A.P., Harris H.M.B., Mattarelli P., O’Toole P.W., Pot B., Vandamme P., Walter J. (2020). A taxonomic note on the genus *Lactobacillus*: Description of 23 novel genera, emended description of the genus *Lactobacillus* Beijerinck 1901, and union of *Lactobacillaceae* and *Leuconostocaceae*. Int. J. Syst. Evol. Microbiol..

[22] Parte A.C., Sardà Carbasse J., Meier-Kolthoff J.P., Reimer L.C., Göker M. (2020). List of prokaryotic names with standing in nomenclature (LPSN) moves to the DSMZ. Int. J. Syst. Evol. Microbiol..

[23] Vandamme P., Pot B., Gillis M., de Vos P., Kersters K., Swings J. (1996). Polyphasic taxonomy, a consensus approach to bacterial systematics. Microbiol. Rev..

[24] Stackebrandt E., Goebel B.M. (1994). Taxonomic note: A place for DNA-DNA reassociation and 16S rRNA sequence analysis in the present species definition in Bacteriology. Int. J. Syst. Bacteriol..

[25] Parks D.H., Imelfort M., Skennerton C.T., Hugenholtz P., Tyson G.W. (2015). CheckM: Assessing the quality of microbial genomes recovered from isolates, single cells, and metagenomes. Genome Res..

[26] Seemann T. (2014). Prokka: Rapid prokaryotic genome annotation. Bioinformatics.

[27] Page A.J., Cummins C.A., Hunt M., Wong V.K., Reuter S., Holden M.T., Fookes M., Falush D., Keane J.A., Parkhill J. (2015). Roary: Rapid large-scale prokaryote pan genome analysis. Bioinformatics.

[28] Cantalapiedra C.P., Hernández-Plaza A., Letunic I., Bork P., Huerta-Cepas J. (2021). eggNOG-mapper v2: Functional Annotation, Orthology Assignments, and Domain Prediction at the Metagenomic Scale. Mol. Biol. Evol..

[29] Huerta-Cepas J., Szklarczyk D., Heller D., Hernández-Plaza A., Forslund S.K., Cook H., Mende D.R., Letunic I., Rattei T., Jensen L.J. (2019). eggNOG 5.0: A hierarchical, functionally and phylogenetically annotated orthology resource based on 5090 organisms and 2502 viruses. Nucleic Acids Res..

[30] Palù M., Basile A., Zampieri G., Treu L., Rossi A., Morlino M.S., Campanaro S. (2022). KEMET—A python tool for KEGG Module evaluation and microbial genome annotation expansion. Comput. Struct. Biotechnol. J..

[31] Zheng J., Ge Q., Yan Y., Zhang X., Huang L., Yin Y. (2023). dbCAN3: Automated carbohydrate-active enzyme and substrate annotation. Nucleic Acids Res..

[32] Oksanen J., Blanchet F.G., Kindt R., Legendre P., O’hara R.B., Simpson G.L., Solymos P., Stevens M.H.H., Wagner H., Barbour M. (2019). vegan: Community Ecology Package. R Package Version 2.5-6. https://CRAN.R-project.org/package=vegan.

[33] Paradis E., Claude J., Strimmer K. (2004). APE: Analyses of Phylogenetics and Evolution in R language. Bioinformatics.

[34] Gosselin S., Fullmer M.S., Feng Y., Gogarten J.P. (2022). Improving Phylogenies Based on Average Nucleotide Identity, Incorporating Saturation Correction and Nonparametric Bootstrap Support. Syst. Biol..

[35] Stamatakis A. (2014). RAxML version 8: A tool for phylogenetic analysis and post-analysis of large phylogenies. Bioinformatics.

[36] Desper R., Gascuel O. (2002). Fast and accurate phylogeny reconstruction algorithms based on the minimum-evolution principle. J. Comput. Biol..

[37] Schliep K.P. (2011). Phangorn: Phylogenetic analysis in R. Bioinformatics.

[38] Huson D.H., Bryant D. (2006). Application of phylogenetic networks in evolutionary studies. Mol. Biol. Evol..

[39] Bandelt H.J., Dress A.W. (1992). Split decomposition: A new and useful approach to phylogenetic analysis of distance data. Mol. Phylogenet. Evol..

[40] Raimondi S., Candeliere F., Amaretti A., Costa S., Vertuani S., Spampinato G., Rossi M. (2022). Phylogenomic analysis of the genus *Leuconostoc*. Front. Microbiol..

[41] Wang Y., Wu J., Lv M., Shao Z., Hungwe M., Wang J., Bai X., Xie J., Wang Y., Geng W. (2021). Metabolism Characteristics of Lactic Acid Bacteria and the Expanding Applications in Food Industry. Front. Bioeng. Biotechnol..

[42] Gänzle M.G. (2015). Lactic metabolism revisited: Metabolism of lactic acid bacteria in food fermentations and food spoilage. Curr. Opinin. Food Sci..

[43] Cogan T.M., Jordan K.N. (1994). Metabolism of *Leuconostoc* bacteria. J. Dairy Sci..

[44] Ren Q., Kang K.H., Paulsen I.T. (2004). TransportDB: A relational database of cellular membrane transport systems. Nucleic Acids Res..

[45] Reque P.M., Pinilla C.M.B., Tinello F., Corich V., Lante A., Giacomini A., Brandelli A. (2020). Biochemical and functional properties of wheat middlings bioprocessed by lactic acid bacteria. J. Food Biochem..

[46] Zafar H., Saier M.H. (2020). Comparative Genomics of the Transport Proteins of Ten *Lactobacillus* Strains. Genes.

[47] Mende S., Rohm H., Jaros D. (2016). Influence of exopolysaccharides on the structure, texture, stability and sensory properties of yoghurt and related products. Int. Dairy J..

[48] Kumar S., Bansal K., Sethi S.K. (2022). Comparative genomics analysis of genus *Leuconostoc* resolves its taxonomy and elucidates its biotechnological importance. Food Microbiol..

[49] Chen Y., Li N., Zhao S., Zhang C., Qiao N., Duan H., Xiao Y., Yan B., Zhao J., Tian F. (2021). Integrated phenotypic–genotypic analysis of *Latilactobacillus sakei* from different niches. Foods.

[50] Sharma A., Sharma N., Gupta D., Lee H.J., Park Y.S. (2022). Comparative genome analysis of four *Leuconostoc* strains with a focus on carbohydrate-active enzymes and oligosaccharide utilization pathways. Comput. Struct. Biotechnol. J..

[51] Frantzen C., Kot W., Pedersen T.B., Ardö Y., Broadbent J.R., Neve H., Hansen L.H., Dal Bello F., Østlie H.M., Kleppen H.P. (2017). Genomic characterization of dairy associated *Leuconostoc* species and diversity of leuconostocs in undefined mixed mesophilic starter cultures. Front. Microbiol..

[52] Yang Y., Feng F., Zhou Q., Zhao F., Du R., Zhou Z., Han Y. (2018). Isolation, purification and characterization of exopolysaccharide produced by *Leuconostoc pseudomesenteroides* YF32 from soybean paste. Int. J. Biol. Macromol..

[53] Wu J., Yan D., Liu Y., Luo X., Li Y., Cao C., Li M., Han Q., Wang C., Wu R. (2021). Purification, Structural Characteristics, and Biological Activities of Exopolysaccharide Isolated from *Leuconostoc mesenteroides* SN-8. Front. Microbiol..

[54] Wang Y., Du R., Qiao X., Zhao B., Zhou Z., Han Y. (2020). Optimization and characterization of exopolysaccharides with a highly branched structure extracted from *Leuconostoc citreum* B-2. Int. J. Biol. Macromol..

[55] Wu Y., Gu C.T. (2021). *Leuconostoc falkenbergense* sp. nov.; isolated from a lactic culture, fermentating string beans and traditional yogurt. Int. J. Syst. Evol. Microbiol..

[56] Jay J.M., Rivers G.M., Boisvert W.E. (1983). Antimicrobial Properties of α-Dicarbonyl and Related Compounds. J. Food. Prot..

[57] García Quintans N., Blancato V., Repizo G., Magni C., López P., Mayo B., López P., Pérez-Martínez G. (2008). Citrate metabolism and aroma compound production in lactic acid bacteria. Molecular Aspects of Lactic Acid Bacteria for Traditional and New Applications.

[58] Laëtitia G., Pascal D., Yann D. (2014). The Citrate Metabolism in Homo- and Heterofermentative LAB: A Selective Means of Becoming Dominant over Other Microorganisms in Complex Ecosystems. Food Nutr. Sci..

[59] Prusova B., Licek J., Kumsta M., Baron M., Sochor J. (2024). Effect of new methods for inhibiting malolactic fermentation on the analytical and sensory parameters of wines. Fermentation.

[60] Schümann C., Michlmayr H., Del Hierro A.M., Kulbe K.D., Jiranek V., Eder R., Nguyen T.H. (2013). Malolactic enzyme from *Oenococcus oeni*: Heterologous expression in *Escherichia coli* and biochemical characterization. Bioengineered.

[61] Montaño A., Sánchez A.H., Casado F.J., Beato V.M., de Castro A. (2013). Degradation of ascorbic acid and potassium sorbate by different *Lactobacillus* species isolated from packed green olives. Food Microbiol..

