# Supplementary material for: The Metabolism of Leuconostoc Genus Decoded by Comparative Genomics
Source: Microorganisms. 2024 Jul 20;12(7):1487. doi: 10.3390/microorganisms12071487 (PMC11279345; doi:10.3390/microorganisms12071487)
Supplement: Supplementary file 1 [file microorganisms-12-01487-s001.zip › Leuconostoc supplementary.pdf]

Supplementary Material

Supplementary Table S1. Nominal taxonomy of the analyzed genomes

Species	No. genomes
L. fallax	4
L. carsosum	20
L. rapi	1
L. kimchii	2
L. miyukkimchii	1
L. gelidum	19
subsp. *gelidum* (14)	
subsp. *aenigmaticum* (4)	
L. gasicomitatum	32
L. holzapfelii	3
L. lactis	30
L. palmae	1
L. citreum	58
L. pseudomesenteroides	15
L. falkenbergense	13
L. litchii	2
L. suionicum	23
L. mesenteroides	219
subsp. *cremoris* (22)	
subsp. *dextranicum* (4)	
subsp. *jonggajibkimchii* (1)	
subsp. *mesenteroides* (21)	
subsp. *sake* (1)	
L. inhae	1
"Leuconostoc garlicum"	1
Leuconostoc sp.	8

Supplementary Table S2. Number of transporters belonging to Major Facilitator Superfamily (MFS), as annotated by NCBI in the genome of *Leuconostoc* type-strains

**Species**	**No. MFS**
L. fallax	28
L. carsosum	28
L. rapi	31
L. kimchii	33
L. miyukkimchii	31
L. gelidum	41
L. gasicomitatum	39
L. holzapfelii	24
L. lactis	19
L. palmae	17
L. citreum	29
L. pseudomesenteroides	32
L. falkenbergense	28
L. litchii	24
L. suionicum	31
L. mesenteroides	33
L. inhae	32

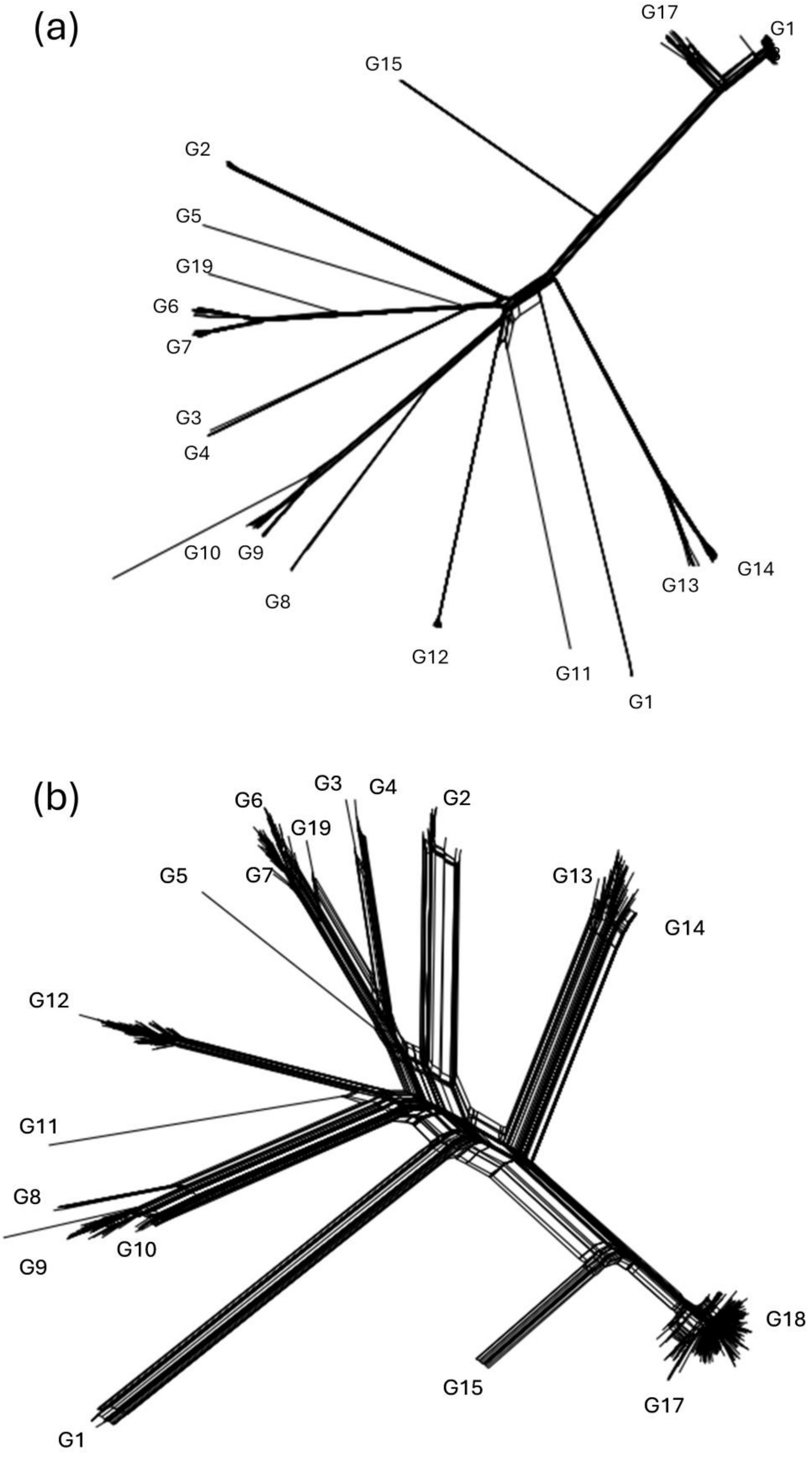

Supplementary Figure S1. Split decomposed phylogenetic trees based on (a) core genes alignment and (b) ANI values.

Colour	Value
(dark green)	100%
(light green)	> 75%
(pale green)	50-75%
(grey)	< 50%
(white)	0%

	dbCAN-sub ID	S.P.	G1	G2	G3	G4	G5	G6	G7	G8	G9	G10	G11	G12	G13	G14	G15	G17	G18	G19
GT2	GT2	N	12	3-8	10	7-9	11	7-12	4-11	7-8	6-10	3-9	5	7-14	8-15	6-13	10	5-11	3-12	10
	GT2+GT2	N	-	-	-	-	-	1	1	-	-	-	-	1	1	-	1	1	1	-
GT4	GT4_e74	N	-	1	-	1	1	1	1	-	-	-	-	-	1	1	-	-	1-2	-
	GT4_e85	N	-	1-2	-	1	1	1	1	-	-	-	-	-	-	1	-	-	1	-
	GT4_e167	N	-	-	-	-	-	-	-	-	-	-	-	-	-	1	-	-	1	1
	GT4_e272	N	1	1	1	1	1	1	1	1	1	1	1	1	1	1	1	1	1	1
	GT4_e332	N	-	-	1	-	-	1	1	-	1	1	-	1	-	1	-	-	1	-
	GT4_e945	N	1	-	1	1	-	1-2	1	1	-	-	1	1	1	-	1	1	-	1
	GT4_e1398	N	-	-	-	-	1	-	-	-	-	-	-	-	-	-	-	-	-	-
	GT4_e1712	N	1	1	1	1	1	1	1	1	1	1	1	1	1	1	1	1	1	1
	GT4_e1908	N	-	-	-	-	-	-	-	-	-	-	-	-	1	1	-	-	-	-
	GT4_e1994	N	1	1	1	1	1	1	1	1	1	1	1	1	2-3	1	1	1	1	1
	GT4_e2015	N	-	-	-	-	-	-	-	-	-	-	-	-	1	1	-	1	1	-
	GT4_e2296	N	-	-	-	-	-	-	-	-	-	-	-	-	-	-	-	1	1	-
	GT4_e2671	N	1	1	-	-	1	1	1	1	1	1	1	1	1	1	1	1	1	1
	GT4_e3978	N	-	-	-	-	-	-	-	-	-	-	-	1	1	1	1	1	1	-
GT8	GT8_e86	N	1	-	1	1	-	1	1	-	-	-	-	-	-	-	-	1	-	-
	GT8_e88	N	-	-	-	1	1	-	-	1	1	1	-	1	-	1	-	1	1-2	1
GT28	GT28_e1	N	1	1	1	1	1	1	1	1	1	1	1	1	1	1	1	1	1	1
GT51	GT51_e10	N	1	1	1	1	1	1	1	1	1	1	1	1	1	1	1	1	1-2	1
	GT51_e50	N	1	1	1	1	1	1	1	1	1	1	1	1	1	1	1	1	1	1
GT83	GT83_e74	N	1	-	-	-	-	-	-	-	-	-	-	-	-	-	1	-	1	-
GT92	GT92_e1	N	-	1	-	-	-	1	1	-	1	1	-	1	1	-	1	1	1	-
GT101	GT101_e0	N	-	-	-	-	-	-	-	1	-	-	-	1	1	-	-	-	-	-
GT111	GT111_e1	N	1	1	1	1	1	1	1	1	1	1	1	1	1	1	-	-	-	1
	GT111_e12	N	-	-	-	-	-	-	-	-	-	-	-	-	1	1	1	1	1	-
GT113	GT113_e13	N	-	-	1	1	-	1	1	1-2	-	-	-	1	1	-	-	-	-	-
	GT113_e16	N	-	-	-	-	-	-	-	-	-	-	1	-	-	-	1	-	-	-
	GT113_e27	N	-	-	-	-	-	1	1	-	-	-	-	-	-	-	-	-	-	1
	GT113_e31	N	-	-	-	-	-	-	-	-	-	-	-	-	-	-	1	1	-	-
	GT113_e35	N	1	1	1	1	1	1	1	1	1	1	1	1	1	1	1	1	1	1

Supplementary Figure S2. Heatmap of GTs presence in *Leuconostoc* groups. S.P. column reports the presence (Y) or absence (N) of the signal peptide. The number (or the range) of genes is given in the cells.
